# Ants Sense, and Follow, Trail Pheromones of Ant Community Members

**DOI:** 10.3390/insects10110383

**Published:** 2019-11-01

**Authors:** Jaime M. Chalissery, Asim Renyard, Regine Gries, Danielle Hoefele, Santosh Kumar Alamsetti, Gerhard Gries

**Affiliations:** Department of Biological Sciences, Simon Fraser University, Burnaby, BC V5A 1S6, Canada; asim_renyard@sfu.ca (A.R.); margret_gries@sfu.ca (R.G.); danielle_hoefele@sfu.ca (D.H.); santoshchem2020@gmail.com (S.K.A.); gerhard_gries@sfu.ca (G.G.)

**Keywords:** *Lasius niger*, black garden ant, *Camponotus modoc*, Western carpenter ant, *Myrmica rubra*, European fire ant, trail pheromone, eavesdropping, pheromonal communication, gas chromatographic-electroantennographic detection

## Abstract

Ants deposit trail pheromones that guide nestmates to food sources. We tested the hypotheses that ant community members (Western carpenter ants, *Camponotus modoc*; black garden ants, *Lasius niger*; European fire ants, *Myrmica rubra*) (1) sense, and follow, each other’s trail pheromones, and (2) fail to recognize trail pheromones of allopatric ants (pavement ants, *Tetramorium caespitum*; desert harvester ants, *Novomessor albisetosus*; Argentine ants, *Linepithema humilis*). In gas chromatographic-electroantennographic detection analyses of a six-species synthetic trail pheromone blend (6-TPB), *La. niger*, *Ca. modoc*, and *M. rubra* sensed the trail pheromones of all community members and unexpectedly that of *T. caespitum*. Except for *La. niger*, all species did not recognize the trail pheromones of *N. albisetosus* and *Li. humilis*. In bioassays, *La. niger* workers followed the 6-TPB trail for longer distances than their own trail pheromone, indicating an additive effect of con- and hetero-specific pheromones on trail-following. Moreover, *Ca. modoc* workers followed the 6-TPB and their own trail pheromones for similar distances, indicating no adverse effects of heterospecific pheromones on trail-following. Our data show that ant community members eavesdrop on each other’s trail pheromones, and that multiple pheromones can be combined in a lure that guides multiple species of pest ants to lethal food baits.

## 1. Introduction

Ant colonies use multimodal communication signals to coordinate specific tasks such as foraging, nest defense, and cooperative brood care [1]. Trail pheromone signals are particularly important in the context of foraging [2]. When a forager has located a profitable food source and then returns to her nest, she deposits trail pheromones that guide nest mates to the same resource [2]. Additional foragers recruited to this resource may also deposit trail pheromones and thus reinforce the original trail [2,3], effectively resulting in collective decisions by nestmates as to which resource to exploit [3,4].

Pheromone trails leading to persistent food sources are generally well maintained by foragers [2] and thus are readily exploited by (heterospecific) non-nestmates [1,5] that learn about the location of profitable food sources through eavesdropping [6,7,8,9]. We use the term “eavesdropping” here to describe the behavior of ants gleaning trail pheromone information from community members but not to imply inevitably adverse effects for any community member involved. Indeed, aggressive encounters of ants with non-nest mates on shared (eavesdropped) trails [1,8,9] are kept to a minimum, in part, by using dissimilar foraging schedules. Temporal partitioning of activity schedules has been reported for workers of Ca. *pennsylvanicus* and *Formica subsericea* that forage on the same aphid-infested trees but at different times of the day [10], and for workers of Ca. *beebei* that follow trails of *Az. charifex* when *Azteca* ants are resting [1,6,8,9]. We anticipate that mutual recognition of pheromone trails is more likely for co-evolved ant species than for native and invasive species. However, two exceptions are conceivable. First, the invasion event took place a long time ago and, over time, the invading species has become a well-established and integrated community member. Second, the invading species is closely related to native species and thus produces a similar trail pheromone.

Ant communities in the Lower Mainland of British Columbia (BC), Canada are complex and comprise both native and invasive species. For the purpose of this study, we have selected three species that co-exist in the same community: (1) the Western carpenter ant, *Camponotus modoc* (Formicinae), which is considered native to the Pacific Northwest and has been recorded in BC as early as 1919 [11]; (2) the black garden ant, *Lasius niger* (Formicinae), which is native to Europe, and possibly to North America, having been recorded in the New World as early as 1979 [11,12,13]; and (3) the European fire ant, *Myrmica rubra* (Myrmicinae), which is native to Europe but has invaded the Pacific Northwest and other parts of North America, likely in the first decade of the 20th century [14,15]. While *Ca. modoc* and *La. niger* have co-existed for at least 39 years [13], *M. rubra* as a more recent adventive is known to have occurred in ant communities of BC’s Lower Mainland for nearly 20 years [15] and has already become a well-established and integrated community member. During 20 years of co-existence, all three species might have “learned” to sense each other’s trail pheromones.

We prepared the trail pheromone components currently known for these three species (*Ca. modoc*: (2*S*,4*R*,5*S*)-2,4-dimethyl-5-hexanolide (henceforth “hexanolide”) [16]; *La. niger*: 3,4-dihydro-8-hydroxy-3,5,7-trimethylisocoumarin (henceforth “isocoumarin”) [17]; *M. rubra*: 3-ethyl-2,5-dimethylpyrazine [18]) in a synthetic blend (Table 1). This blend also contained 3-ethyl-2,6-dimethyl pyrazine (a non-natural isomer in the commercial source of the *M. rubra* trail pheromone). To determine whether *Ca. modoc*, *La. niger*, and *M. rubra* sense the trail pheromones not only of community members but also of allopatric ant species, we expanded the synthetic blend to include the trail pheromone of the pavement ant, *Tetramorium caespitum* (2,5-dimethylpyrazine), the desert harvester ant, *Novomessor albisetosus* (4-methyl-3-heptanone), and the Argentine ant, *Linepithema humilis* ((*Z*)-9-hexadecenal) [19,20,21].

Here, we tested the hypotheses that sympatric *Ca. modoc*, *La. niger*, and *M. rubra* (1) sense, and behaviorally respond to, each other’s trail pheromones, and (2) fail to recognize the trail pheromones of allopatric ant species (*T. caespitum*, *N. albisetosus*, *Li. humilis*).

## 2. Materials and Methods

### 2.1. Experimental Insects

#### 2.1.1. Lasius Niger

Between 13 and 31 August (2018), 5–10 ants were collected in various containers from each of 20 sites throughout Vancouver and Burnaby, BC. Ants were bioassayed within 24 h of collection, and then cold-euthanized for taxonomic confirmation using multiple keys [13,22,23,24].

#### 2.1.2. Camponotus Modoc

Collection and maintenance of *Ca. modoc* nests have recently been described in detail [16]. Briefly, infested log sections were kept in large plastic bins (64 cm × 79 cm × 117 cm) in an outdoor undercover area exposed to natural light and temperature cycles throughout the year. Each plastic bin housing a nest was connected via clear PVC tubing (2.54 cm I.D., Nalgene™ 180; Sigma-Aldrich, St. Louis, MO, USA) to a glass aquarium (51 × 28 × 30 cm), which served as the ants’ foraging area provisioned with blow flies, live mealworms, honey, apples, canned chicken, and 20% sugar water, all *ad libitum*.

#### 2.1.3. Myrmica Rubra

In the spring and summer of 2017 and 2018, 20 nests of *M. rubra* were dug out of the ground at Inter River Park (North Vancouver, BC, Canada), the Regional Allotment Garden (Burnaby, BC, Canada), and the VanDusen Botanical Garden (Vancouver, BC, Canada). Nests were kept indoors in the Science Research Annex of Simon Fraser University (49°16′33″ N, 122°54′55″ W) at 25 °C and a photoperiod of 12 h L to 12 h D. Nests were housed in small Tupperware dishes (15 × 15 × 9 cm) (Rubbermaid^®^, Newell Brands, Atlanta, GA, USA & Royal Sponge Manufacturing Ltd., Toronto, ON, Canada), which were fitted with sterilized potting soil as nesting material and placed inside a small or large tote (41 × 29 × 24 cm; 58 × 43 × 31 cm) that served as the ants’ foraging area. Twice a week, the nests were sprayed with water and provisioned with food (fruits, nuts, mealworms, and processed meat). Test tube water reservoirs were replaced when low.

### 2.2. Gas Chromatographic-Electroantennographic Detection (GC-EAD) Analyses of Synthetic Ant Trail Pheromones

For GC-EAD analyses and behavioral bioassays, a synthetic blend of six ant trail pheromones (see above), henceforth six-trail pheromone blend (6-TPB; Table 1), was prepared. The blend was analyzed by gas chromatographic-electroantennographic detection (GC-EAD), with procedures and equipment previously described in detail [25,26]. Briefly, the GC-EAD setup employed a Hewlett-Packard 5890 gas chromatograph (GC) fitted with a DB-5 GC column (30 m × 0.32 mm I.D.; J&W Scientific, Folsom, CA, USA). Helium served as the carrier gas (35 cm·s^−1^) with the following temperature program: 50 °C for 1 min, 20 °C·min^−1^ to 280 °C. The injector port and flame ionization detector (FID) were set to 260 °C and 280 °C, respectively. For GC-EAD recordings (three antennae each for *Ca. modoc*, *La. niger*, and *M. rubra*), an antenna was carefully dislodged from a worker ant and suspended between two glass capillary electrodes (1.0 × 0.58 × 100 mm; A-M Systems, Carlsborg, WA, USA) prepared to accommodate the antenna and filled with a saline solution [27].

### 2.3. General Design of Trail-Following Bioassays

**Figure 1:**
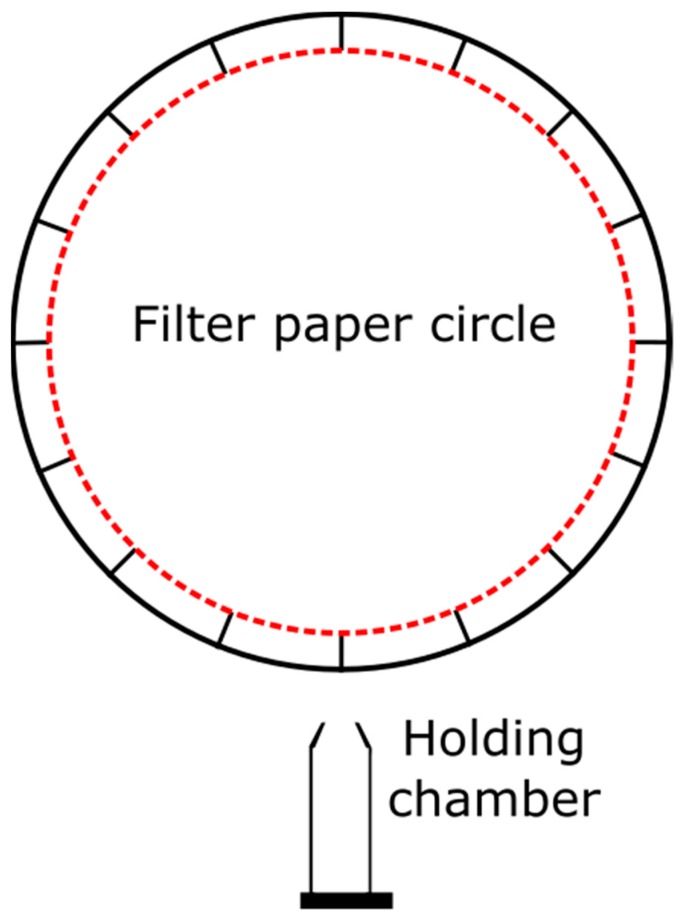
Graphical illustration of the experimental design used for testing trail-following of Western carpenter ants, *Camponotus modoc*, black garden ants, *Lasius niger*, and European fire ants, *Myrmica rubra*, in response to their own trail pheromone or a complex blend of six trail pheromones (see Table 1). To account for body size differentials of large ants (*Ca. modoc*) and small ants (*La. niger*; *M. rubra*), bioassay arenas were large (64 cm wide × 44 cm long × 10 cm high) or small (circular, 15 cm diam × 1 cm high), and the diameter of the filter paper was 18.5 and 9 cm, respectively. Pheromone trails were applied to the filter paper along the red dotted line (which was absent in bioassays).

All bioassays were run within a metal scaffold (123 × 57 × 36 cm) encased in black fabric to eliminate external visual stimuli, lit from above with two fluorescent lights (48″ 32 W F32T8, one plant and aquarium bulb, and one daylight bulb, Phillips, Amsterdam, The Netherlands), and fitted with a video camera (Sony HDR CX210, Sony, Tokyo, Japan or Canon FS100 A, Canon, Tokyo, Japan) mounted above the bioassay arena (Figure 1). The edges of the bioassay arenas (see below) were coated with a mixture of petroleum jelly and mineral oil to prevent the escape of bioassay ants.

The specific experimental design to test trail-following responses accounted for body size differentials of large ants (*Ca. modoc*) and small ants (*La. niger* and *M. rubra*). The design for testing *Ca. modoc* was previously described [16] and is outlined here. *Camponotus modoc* was tested in a large plexiglass arena (64 × 44 × 10 cm) fitted with a filter paper (18.5 cm diam; Sigma-Aldrich, St. Louis, MO, USA), with its circular circumference marked with pencil in 1 cm intervals (58 marks total) and treated with one of three test stimuli (see below) at 1–2 ant equivalents (AE)/58 µL. Each bioassay ant (n = 60) entered the arena by exiting a 15 mL Falcon™ “holding” tube (Thermo Fisher Scientific, Waltham, MA, USA) through a hole cut in its tapered tip.

*Lasius niger* and *M. rubra* were tested in a Pyrex petri dish (15 cm diam) fitted with a small circular filter paper (9.0 cm diam), with its circumference marked with pencil in 1 cm intervals (25 total) and treated with one of three test stimuli (see below) at 1–2 AEs/25 µL. Each worker ant of *La. niger* (n = 60) and *M. rubra* (n = 60) entered the Petri dish by exiting a 1.5 mL Axygen™ MaxyClear Snaplock “holding” microtube (Thermo Fisher Scientific, Waltham, MA, USA) through a hole cut in its tapered tip. Bioassays of large and small ants were initiated by removing the cotton plug from the exit hole of a holding tube and were terminated after 5 min (*Ca. modoc*) and 10 min (*La. niger* and *M. rubra*). Filter papers were prepared for bioassays by applying a continuous trail of test stimulus [(*i*) synthetic 6-TPB; (*ii*) synthetic trail pheromone of the bioassay ant; or (*iii*) a solvent control; Table 1].

The number of 1 cm intervals an ant had followed during a bioassay served as the response criterion and was analyzed by viewing the video footage. Ants not leaving their holding tube after 10 min were considered non-responders and excluded from analyses. Between bioassays, all preparative surfaces and bioassay arenas were cleaned with 70% EtOH and hexane, and the experiment room was aired out for 5 to 10 min by opening an exterior door. A new ant was tested for each treatment. All *La. niger* and *M. rubra* ants were collected from different laboratory or field colonies. Worker ants of *Ca. modoc* were collected from six colonies maintained in an outdoor enclosure.

### 2.4. Statistics

R (Version 3.5.0; multicomp, plotrix, & plyr packages) was used to analyze the data and produce graphics [28,29,30]. A generalized linear model (GLM; quasi-Poisson distribution) was used to analyze the distances ants travelled following trails in response to the various types of trails presented. An analysis of variance (ANOVA) and Tukey’s honest significant difference (HSD) test were used to determine significant differences in mean distance travelled in response to trail type.

## 3. Results

### 3.1. Gas Chromatographic-Electroantennographic Detection (GC-EAD) Analyses of Synthetic Ant Trail Pheromones

**Figure 2:**
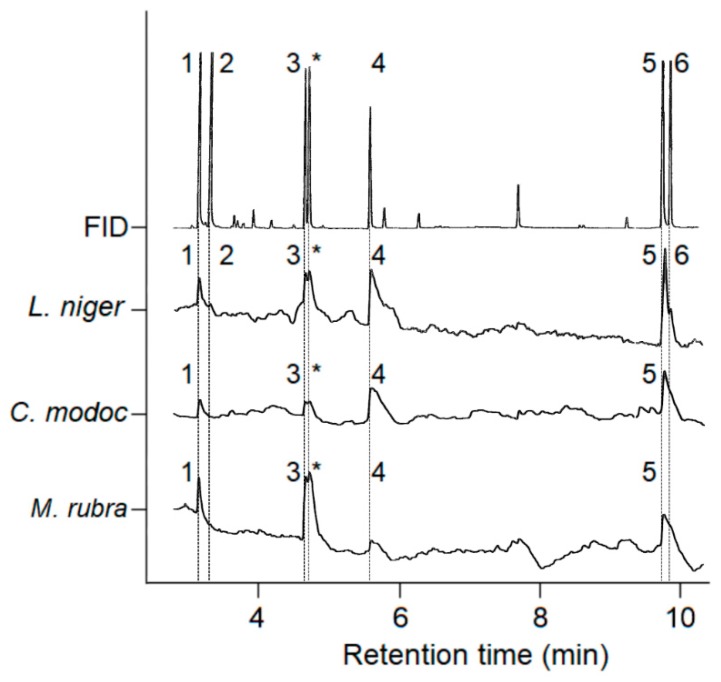
Representative recordings (n = 3 each) of the responses of a gas chromatographic flame ionization detector (FID) and an electroantennographic detector (EAD: Antenna of a *Lasius niger*, *Camponotus modoc*, or *Myrmica rubra* worker ant) to pheromone components present in the six-trail pheromone blend (see Table 1). Numbers in the FID trace refer to the following pheromone components: (1) 2,5-dimethylpyrazine; (2) 4-methyl-3-heptanone; (3) 3-ethyl-2,5-dimethylpyrazine; * = 3-ethyl-2,6-dimethyl pyrazine (non-natural isomer present in synthetic source); (4) (2*S*,4*R*,5*S*)-2,4-dimethyl-5-hexanolide; (5) 3,4-dihydro-8-hydroxy-3,5,7-trimethylisocoumarin; and (6) (*Z*)-9-hexadecanal.

In GC-EAD analyses, *La. niger* antennae responded to (in the order of elution) synthetic 2,5-dimethylpyrazine, 4-methyl-3-heptanone, 3-ethyl-2,5-dimethylpyrazine, 3-ethyl-2,6-dimethyl pyrazine, hexanolide, isocoumarin (its own trail pheromone), and (Z)-9-hexadecenal (Figure 2; Table 2). Antennae of *Ca. modoc* responded to 2,5-dimethylpyrazine, 3-ethyl-2,5-dimethylpyrazine, 3-ethyl-2,6-dimethyl pyrazine, hexanolide (its own trail pheromone), and isocoumarin (Figure 2; Table 2). Antennae of *M. rubra* responded to 2,5-dimethylpyrazine, 3-ethyl-2,5-dimethylpyrazine (its own trail pheromone), 3-ethyl-2,6-dimethyl pyrazine, hexanolide, and isocoumarin (Figure 2; Table 2).

### 3.2. Trail-Following Bioassays

**Figure 3:**
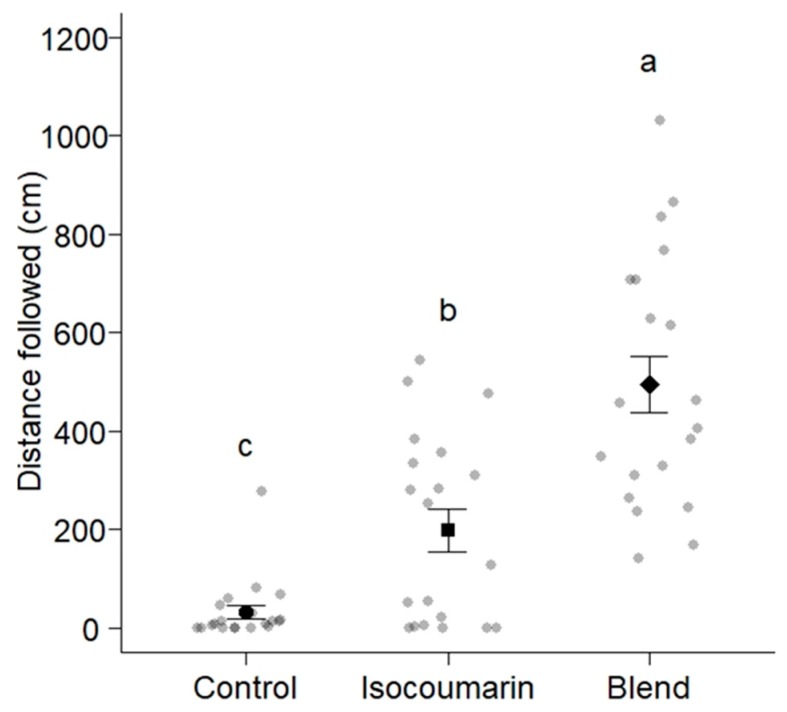
Distances worker ants of *Lasius niger* (n = 60) travelled following trails of synthetic 3,4-dihydro-8-hydroxy-3,5,7-trimethylisocoumarin (the known trail pheromone of *La. niger* [17]), a six-trail pheromone blend (Table 1), and a solvent control applied to the circumference of a circular filter paper (diam: 90 mm) marked in 1-cm intervals (Figure 1). Grey and black symbols show the distance that each ant and 20 ants on average (mean ± whiskers), respectively, travelled following trails. Means associated with different letters are statistically different (Tukey’s honest significant difference (HSD) test, *p* < 0.01); six out of 66 ants tested did not enter the bioassay arena and were excluded from this data set.

There were significant differences in the distances (mean ± SE) that worker ants of *La. niger* travelled following trails of the 6-TPB (495.5 ± 57.5 cm), the isocoumarin (199.3 ± 43.1 cm), and the solvent control (32.2 ± 14.0 cm) (ANOVA, F = 34.028, degrees of freedom (df) = 2, residual df = 57, *p* < 0.001; Figure 3). Based on Tukey’s HSD tests, all distances differed from one another (pairwise comparisons: Isocoumarin vs. solvent control: *p* = 0.001; 6-TPB vs. solvent control: *p* < 0.001; 6-TPB vs. isocoumarin: *p* < 0.001).

**Figure 4:**
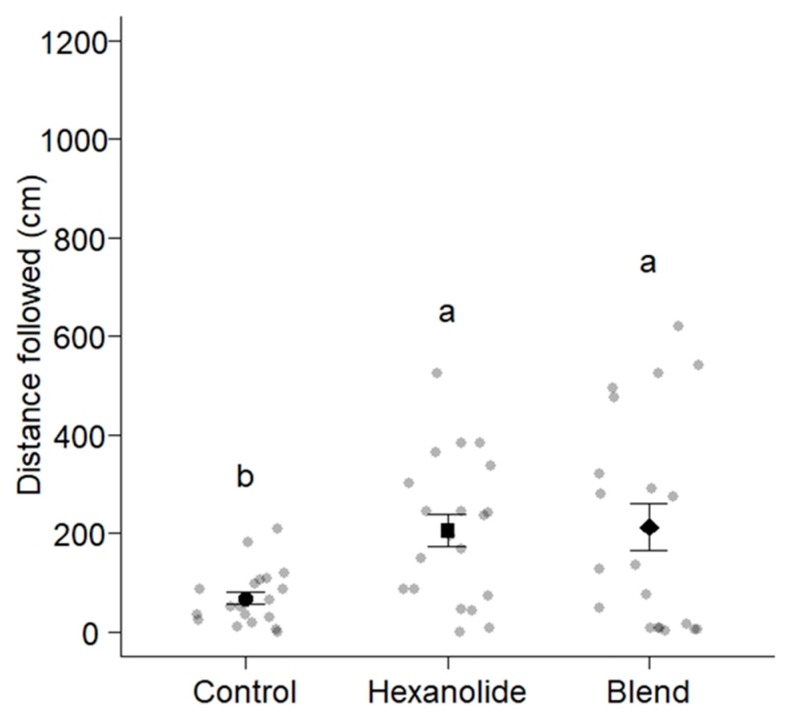
Distances worker ants of *Camponotus modoc* (n = 60) travelled following trails of synthetic (2*S*,4*R*,5*S*)-2,4-dimethyl-5-hexanolide (the known trail pheromone of *Ca. modoc* [16]), a six-trail-pheromone blend (Table 1), and a solvent control applied to the circumference of a circular filter paper (diam: 185 mm) marked in 1 cm intervals (Figure 1). Grey and black symbols show the distance that each ant and 20 ants on average (mean ± whiskers) travelled, respectively, following trails. Means associated with different letters are statistically different (Tukey’s honest significant difference (HSD) test, *p* < 0.01); two out of 62 ants tested did not enter the bioassay arena and were excluded from this data set.

There were also significant differences in the distances that worker ants of *Ca. modoc* travelled following trails of the 6-TPB (213.1 ± 48.7 cm), the hexanolide (206.0 ± 32.7 cm), and the solvent control (69.1 ± 12.6 cm) (ANOVA, F = 7.5583, df = 2, residual df = 57, *p* < 0.01; Figure 4). Based on Tukey’s HSD tests, distances were different between the 6-TPB and the solvent control (*p* < 0.01), and between the hexanolide and the solvent control (*p* < 0.01), but were statistically the same between the 6-TPB and the hexanolide (*p* = 0.99).

**Figure 5:**
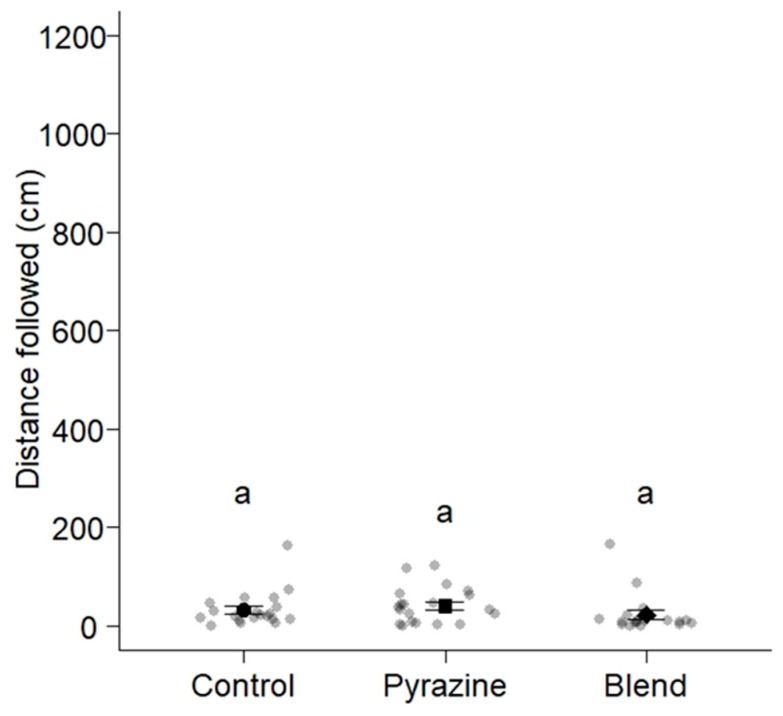
Distances worker ants of *Myrmica rubra* (n = 60) travelled following trails of synthetic 3-ethyl-2,5-dimethylpyrazine (the known trail pheromone of *M. rubra* [19]), a six-trail-pheromone blend (Table 1), and a solvent control applied to the circumference of a circular filter paper (diam: 90 mm) marked in 1-cm intervals (Figure 1). Grey and black symbols show the distance that each ant and 20 ants on average (mean ± whiskers) travelled, respectively, following trails. Means associated with different letters are statistically different (Tukey’s honest significant difference (HSD) test, *p* < 0.01).

There were no significant differences in the distances that worker ants of *M. rubra* travelled following trails of the 6-TPB (22.9 ± 8.7 cm), the 3-ethyl-2,5-dimethylpyrazine (41.2 ± 8.3 cm), and the solvent control (32.9 ± 8.1 cm) (ANOVA, F = 1.1555, df = 2, residual df = 57, *p* = 0.3222; Figure 5).

## 4. Discussion

As predicted, *La. niger*, *Ca. modoc*, and *M. rubra* did sense (antennally respond to) the trail pheromone of all community members (*La. niger*, *Ca. modoc*, *M. rubra*; Figure 2) and, except for *La. niger*, did not recognize the trail pheromones of two allopatric ant species (*N. cockerelli* and *Li. humilis*; Table 2). That all three ant species sensed the trail pheromone of allopatric *T. caespitum* could be due its molecular structure (2,5-dimethylpyrazine) resembling that of the *M. rubra* trail pheromone (3-ethyl-2,5-dimethylpyrazine). In light of our behavioral data that the 6-TPB (which contains trail pheromones of con- and hetero-specifics) readily induced trail-following behavior of *Ca. modoc* and *La. niger*, it seems that these ants either simply ignore (*Ca. modoc*), or indeed eavesdrop on (*La. niger*), each other’s trail pheromone communication. In general, eavesdropping ants can face aggression, increased competition, or even displacement [1,6,8,9,10], but in the ant community we studied here, eavesdropping may accrue more benefits than harm, or at least, no harm. This inference is based on our bioassay data showing that (*i*) *La. niger* workers followed trails of the 6-TPB for a longer distance than they followed their own trail pheromone (isocoumarin), and (*ii*) *Ca. modoc* workers followed trails of the 6-TPB and their own pheromone (hexanolide) for similar distances.

Unexpectedly, workers of *M. rubra* followed trails of the 6-TPB and their own trail pheromone (3-ethyl-2,5-dimethylpyrazine) only as much as a solvent control trail, demonstrating no effect of the trail pheromone in this type of bioassay. There are at least two explanations why *M. rubra* did not follow a trail of synthetic 3-ethyl-2,5-dimethylpyrazine. Prior studies that demonstrated distinct trail following by *M. rubra*, either in “no-choice bioassays” comparable to our experimental design [18] or in “binary-choice arena bioassays” [31], tested the responses of multiple workers (the entire nest), whereas we tested the responses of individual ants. Given the small foraging range and high nest density of *M. rubra* in North America [31,32], it is conceivable that nest mates do not forage on their own but engage in group foraging, as shown in many *Myrmica* species [33]. Group foraging entails cooperative interactions, where, for example, a successful forager recruits nestmates and physically guides them to the food source [33]. Group foraging may improve the overall foraging effort of a nest and facilitate transport of food particles that are too heavy for single ants to carry [34]. Alternatively, the trail pheromone blend of *M. rubra* comprises not only 3-ethyl-2,5-dimethylpyrazine but additional pheromone components, which, thus far, have eluded identification.

The evidence presented here that some ant community members eavesdrop on and exploit each other’s trail pheromone has major implications for ant control. Food baits laced with lethal agents show promise as an ant control tactic because many ants share food through trophallaxis and thus may spread the poison together with the food throughout their entire nest. The effect of lethal food baits can be enhanced by adding attractants. For example, the admixture of trail pheromone to food baits increased bait consumption by the invasive Argentine ant, *L. humile* [35]. In *M. rubra*, a path of synthetic trail pheromone leading from a nest to a food bait is more effective in recruiting foragers than applying the trail pheromone around a food bait [31].

Commercial development of trail pheromones for ant control is contingent upon economic feasibility. With so many important ant species in need of control, and with each species producing its own trail pheromone, manufacturing species-specific (single target) trail pheromone lures (ropes, strings) does not seem economically viable. However, if trail pheromones of multiple ant species were to be combined in a single lure (multiple targets), with potential synergism and no antagonism between components, as shown in our study, then an ant control tactic that couples a lethal food bait with a trail pheromone lure seems commercially feasible. As an added advantage, the ant species targeted for control would not even need to be identified by a pest control professional or the lay person buying the control technology in a retail store.

Future studies should aim to strengthen the proof of concept presented in our study. Trail pheromones of major ant pests such as the red imported fire ant, *Solenopsis invicta*, should be added to the multiple-species trail pheromone lure and tested for the response of *S. invicta* and other species. Moreover, research needs to be initiated on dispensers capable of sustained release of trail pheromones in field experiments and, eventually, operational applications.

## 5. Conclusions

All three select members of ant communities in the Lower Mainland of BC (*La. niger*, *Ca. modoc*, *M. rubra*) sensed each other’s trail pheromone and, except for *La. niger*, did not recognize the trail pheromones of two allopatric ant species (*N. cockerelli* and *Li. humilis*). Workers of *La. niger* followed a synthetic trail pheromone blend (containing the trail pheromone of all three community members and those of three allopatric ant species) for a longer distance than they followed their own trail pheromone, and *Ca. modoc* workers followed this blend and their own trail pheromone for similar distances. Apparently, these ants either ignore (*Ca. modoc*), or indeed eavesdrop on (*La. niger*), each other’s trail pheromone. Eavesdropping ants may accrue benefits by learning about the location of profitable food sources. If synthetic trail pheromones of multiple pest ant species were to be combined in a single (rope-type) lure, with potential synergism and no antagonism between components (as shown in our study), an ant control tactic that presents a lethal food bait together with a trail pheromone lure seems commercially viable.

## Figures and Tables

**Table 1:** List of trail pheromones (and select species producing them) comprising the six-trail pheromone blend (6-TPB) tested in circular trail bioassays (Figure 1) and in electrophysiological recordings (Figure 2, Table 2). In trail bioassays (Figure 3, Figure 4 and Figure 5), the trail-following of *Camponotus modoc*, *Lasius niger*, and *Myrmica rubra* was each tested in response to (*i*) the 6-TPB formulated in pentane, (*ii*) their own trail pheromone formulated in pentane, and (*iii*) a pentane control. Stimuli were tested at 1–2 ant equivalents (AE)/58 µL (*Ca. modoc*) and 1–2 AEs/25 µL (*La. niger* and *M. rubra*) to account for the length differential of stimulus trails that were tested for large ants (*Ca. modoc*) and small ants (*La. niger* and *M. rubra*) (see Methods for detail).

Study Species	Name of Pheromone (Amount Tested; Ant Equivalents (AEs))	Pheromone Structures (Synthetic Sources a–e)
*Ca. modoc*	(2*S*,4*R*,5*S*)-2,4-Dimethyl-5-hexanolide (7.5 ng; 2 AEs) (“hexanolide”) [16]	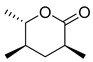 (a)
*La. niger*	3,4-Dihydro-8-hydroxy-3-7-trimethylisocoumarin (0.5 ng; 1 AE) (“isocoumarin”) [17]	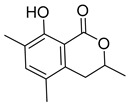 (a)
*M. rubra*	3-Ethyl-2,5-dimethylpyrazine (5 ng; 1 AE) [18]	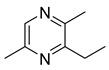 (b)
*T. caespitum*	2,5-Dimethylpyrazine (1 ng; 1 AE) [19]	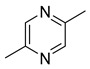 (c)
*N. cockerelli*	4-Methyl-3-heptanone (10 ng;1 AE) [20]	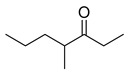 (d)
*Li. humilis*	(*Z*)-9-Hexadecenal (10 ng; 1 AE) [21]	CH_3_-(CH_2_)_4_-CH=CH-(CH_2_)_7_-CHO (e)

(a) Synthesized as described by Renyard et al. [16]; (b) Acros Organics, New Jersey, USA (contains 3-ethyl-2,5-dimethylpyrazine at 50%); (c) Aldrich Chem Co. Milwaukee, WI, USA; (d) oxidized from 4-methyl-3-heptanol (Sigma-Aldrich, St. Louis, MO, USA); (e) oxidized from (*Z*)-9-hexadecenol (Sigma-Aldrich).

**Table 2:** List of trail pheromone components produced by sympatric ant species inhabiting ant communities in the Pacific Northwest (Western carpenter ants, *Camponotus modoc*; black garden ants, *Lasius niger*; European fire ants, *Myrmica rubra*) and by allopatric ant species (pavement ants, *Tetramorium caespitum*; desert harvester ants, *Novomessor albisetosus*; Argentine ants, *Linepithema humilis*), as well as information as to whether community members (*La. niger*, *Ca. modoc*, and *M. rubra*) antennally respond to these components in electrophysiological recordings (summary of gas chromatographic-electroantennographic detection (GC-EAD) results; see Figure 2).

Distribution	Species	Trail Pheromone	Produced by/Antennal Response		
			*La. niger*	*Ca. modoc*	*M. rubra*
*Sympatric*	*La. niger*	Isocoumarin *	yes/yes	yes/yes	no/yes
	*Ca. modoc*	Hexanolide **	no/yes	yes/yes	no/yes
	*M. rubra*	3-Ethyl-2,5-dimethylpyrazine	no/yes	no/yes	yes/yes
*Allopatric*	*T. caespitum*	2,5-Dimethylpyrazine	no/yes	no/yes	no/yes
	*N. albisetosus*	4-Methyl-3-heptanone	no/yes	no/no	no/no
	*Li. humilis*	(*Z*)-9-Hexadecenal	no/yes	no/no	no/no

* 3,4-Dihydro-8-hydroxy-3,5,7-trimethylisocoumarin; ** 2,4-Dimethyl-5-hexanolide.
